# Cells with Stem Cell Characteristics in Somatic Compartments of the Ovary

**DOI:** 10.1155/2013/310859

**Published:** 2012-12-27

**Authors:** Katarzyna Kossowska-Tomaszczuk, Christian De Geyter

**Affiliations:** Clinic of Gynecological Endocrinology and Reproductive Medicine and Department of Biomedicine, University of Basel, 4031 Basel, Switzerland

## Abstract

Antral follicular growth in the ovary is characterized by rapid expansion of granulosa cells accompanied by a rising complexity of their functionality. Within two weeks the number of human granulosa cells increases from less than 500,000 to more than 50 millions cells per follicle and differentiates into groups of cells with a variety of specialized functions involved in steroidogenesis, nursing the oocyte, and forming a functional syncitium. Both the rapid proliferation and different specialized functions of the granulosa cells can only be explained through the involvement of stem cells. However, luteinizing granulosa cells were believed to be terminally differentiated cells. Only recently, stem and progenitor cells with FSH-receptor activity were identified in populations of luteinizing granulosa cells obtained during oocyte collected for assisted reproduction. In the presence of the leukaemia-inhibiting factor (LIF), it was possible to culture a subpopulation of the luteinizing granulosa cells over prolonged time periods. Furthermore, when embedded in a matrix consisting of collagen type I, these cells continued to express the FSH receptor over prolonged time periods, developed globular formations that surrogated as follicle-like structures, providing a promising tool for reproductive biology.

## 1. The Dynamics of Ovarian Follicular Growth and Development

Ovarian follicular development is initiated from a pool of inactive primordial follicles. Each follicle contains a small nongrowing oocyte and a single layer of nondividing cells encapsulated by the follicular basal lamina [1]. As part of an ongoing process, primordial follicles become active, the oocyte starts to grow, and its surrounding granulosa cells start to become mitotic. As the granulosa cells divide, the number of layers of granulosa cells (called the membrane granulosa or follicular epithelium) around the oocyte increases, and the basal lamina expands [2, 3]. Primordial follicles give rise to primary follicles which transform into preantral (secondary follicles), then antral follicles (tertiary follicles), and finally preovulatory and Graafian follicles, in a coordinated series of transitions regulated by hormones and local intraovarian factors [1, 4, 5]. During the ultimate stages of follicular growth several millions of GCs [6] exert a multitude of specialized functions encompassing the function of the follicle, such as producing large amounts of estradiol, adapting its FSH, luteinizing hormone receptivity to the endocrine milieu, nursing the oocyte, and communicating both with the enclosed oocyte and the surrounding thecal cells. The signalling leading to ovulation results in luteinization of the remnants of the ovulated follicle. Luteinized GCs are considered to be terminally differentiated, being replaced in the midluteal phase of the menstrual cycle by small, luteinized cells originating from the surrounding theca [7].

The mammalian ovary produces mature oocytes capable of being fertilized and sustaining early embryonic development. Developmental competence of the oocyte correlates with follicular size, larger oocytes being more developmentally competent [8, 9]. The development of an oocyte ultimately capable of undergoing fertilization and embryogenesis depends on appropriate signalling from surrounding ovarian granulosa cells (GCs) and follicle-stimulating hormone (FSH) [10].

## 2. The Role of Surrounding Somatic Cells in Folliculogenesis

There are three ovarian functional somatic cell types involved in folliculogenesis: (1) the ovarian surface epithelium that surrounds ovary, (2) the theca, and (3) the granulosa cells, which essentially reside within the avascular space of the ovarian follicle.

Primordial follicles are not distributed uniformly in the ovary but are predominantly located in the ovarian cortex. The ovarian cortex is covered by a layer of irregularly shaped cells [11], commonly known as the ovarian “germinal” or surface epithelium, which is attached to the tunica albuginea. In functional human ovaries the surface epithelium is found in certain areas only, but in women with polycystic ovaries, the ovarian surface is completely covered with surface epithelium [12]. These observations indicate that the surface epithelium derived epithelial nests may represent primitive granulosa cells. They may either invade surface epithelium from adjacent structures and are extruded from the ovary [13].

The tunica albuginea is a thick fibrous subepithelial layer with cells embedded in connective tissue, which does not begin to form until the end of intrauterine life [13, 14]. In adult human females, mesenchymal cells in the ovarian tunica albuginea undergo a mesenchymal-epithelial transition into ovarian surface epithelial cells (OSE) [13, 15, 16], which may differentiate sequentially into primitive granulosa. These structures assemble in the deeper ovarian cortex may form new follicles to replace earlier primary follicles undergoing atresia [17–19], but this concept still needs verification. 

Theca cells surround the developing follicle, form the two layers known as the theca externa and interna, and produce the androgens which are ultimately converted to estradiol by the GCs.

During the final stages of follicular growth, GCs are at the centre of ovarian function, as they not only direct the growth of the oocytes thereby inhibiting meiotic progress but also produce and secrete the hormones which prepare such processes as ovulation and endometrial proliferation. The antral growth, the proliferation, differentiation, and function of GCs are initially controlled by the follicle-stimulating hormone (FSH) alone, later by both FSH and luteinizing hormone (LH). FSH targets its receptor (FSHR) and induces the maturation of ovarian follicles through proliferation of GCs; induction of the LH-receptor (LHR) and formation of a functional syncytium [20–23] which surrounds and nurses the oocyte produce the bulk of steroids. Steroids are then secreted into blood circulation to manage successful ovulation, fertilization, and subsequent implantation of the embryo. 

The membrana granulosa or follicular epithelium is more complex than most other epithelia for various reasons. At first it expands from a single to a multilayered epithelium, as the follicle grows. In the transition from a pre- to a postantral follicle, the shape of GC changes from nondividing flattened appearance to dividing cubical appearance. The epithelium also expands laterally with time, as the follicle enlarges. During the preovulatory phase the membrana granulosa becomes vascularised with capillaries sprouting from the surrounding theca interna. Finally, at ovulation GCs differentiate into luteal cells. It is the fate of 99% of all follicles to become atretic, and apoptosis among the GCs is one of the first indicators of follicular atresia [2, 9, 24].

## 3. Ovarian Surface Epithelial for Renewal of the Follicle Pool after Birth

Oogenesis has been demonstrated in cultured mouse embryonic stem cells [25], and mitotically active germ cells have been reported in ovaries of adult prosimian primates [26] and mice [27, 28]. Regarding follicular renewal in adult human females, reports provide evidence that the OSE could be a source of germ cells, and new primary follicles are formed by assembly of oocytes with nests of primitive granulosa cells in the ovarian cortex [18, 19]. Components for the new primary follicles, primitive granulosa and germ cells, are proposed to differentiate *de novo* from mesenchymal progenitor cells residing in the ovarian tunica albuginea. During differentiation into OSE cells the mesenchymal progenitor cells line either the ovarian surface or invaginated epithelial crypts. Mesenchymal progenitor cells would first contribute to the development of epithelial cells similar to granulosa cells, and these cells subsequently form epithelial nests descending into the deeper ovarian cortex. These cells may be a source of germ cells, which assemble together with nests of primitive granulosa cells to form primary follicles [17]. Oogenesis may follow later. These reports all represent challenges to established dogma on the fetal origin of mammalian follicles [29, 30].

Gene expression profiling of human ovarian surface epithelial cells suggests that some of these cells are multipotent [31] and these findings are consistent with the hypothesis that OSE expresses many genes involved in somatic stem cell maintenance. Pluripotent stem cells were also found in the OSE of patients with premature ovarian failure [15, 32, 33]. These cells were an integral part of the ovarian surface epithelium and displayed morphology of oocyte cells and expression pattern of pluripotent stem cells.

Two distinct populations of putative stem cells (PSCs) were also detected in scraped OSE [16]: embryonic-like PSCs were pluripotent and underwent spontaneous differentiation into oocyte-like structures, whereas epithelial cells, probably the tissue progenitor stem cells, transformed into mesenchymal phenotype by epithelial-mesenchymal transition.

## 4. Culture of Human Granulosa Stem and Progenitor Cells *In Vitro*

Though GCs are deeply involved in human ovarian function and its various dysfunctions, and little has been known, most likely due the impossibility to culture them over prolonged time periods *in vitro*. Most studies on ovarian functions have been carried out with subhuman primates and nonprimate animals [34] and result from short-term cultures *in vitro*. Existing immortalized human granulosa cell lines, obtained from developing follicles or ovarian carcinomas, showed little steroid hormone biosynthesis and/or limited detectable expression of the genes characteristics for GCs markers [34–38]. Immortalized human GC lines are useful for study follicular and oocyte maturation *in vitro*; however, those lines are not physiological, as most of them were established from a primary human GC tumor or were established by transfection of luteinizing GC.

Only recently methods have been developed to culture luteinizing GCs over prolonged time periods [39]. These cells are available in large quantities, as then can be retrieved from infertile women undergoing controlled ovarian hyperstimulation for assisted reproduction. This source of GCs also carries the advantage, as the donors of these cells are usually well characterized.

The crucial difference between earlier trials was the use of LIF, a cytokine commonly used in culture media supporting the development and growth of stem cells. LIF promoted the long-term survival of luteinizing GC; whereas in the absence of LIF, these cells invariably became apoptotic. LIF is a glycoprotein with a remarkable range of biological actions in different tissues, such as long-term maintenance of mouse, but not human embryonic stem cells [40]. In a number of tissues LIF has been shown to be important for stem cell self-renewal, such as the brain [41], the gut [42], and bone marrow [43].

LIF has been detected both in fetal and adult human ovaries [44], is present in the follicular fluid, and may be involved in the transition of primordial to primary follicles [45].

In addition to the specific effects of the various components of the ECM, the latter allows the cells to grow in a 3D environment, which has been shown to be essential for sustaining the morphology of ovarian follicles, including cell-cell and cell-matrix interactions [4, 46], thereby promoting follicular growth and cell proliferation. In a 2D culture system, murine follicles fail to maintain their *in vivo*-like architecture [9, 47, 48] and typically fail to grow [49]. The short-term beneficial effects of culturing GC in a 3D environment have been demonstrated previously [50, 51], but not the long-term effects. 

Alginate hydrogels, a widely used substitute of the ECM in tissue engineering and characterized by optimal biomechanical properties, have been used to promote the development of mouse ovarian follicles *in vitro*, and both oocyte maturation and life offspring have already been achieved with this method [48, 52]. However, alginate hydrogels are manufactured from brown algae and consist of a polymeric scaffold of polysaccharides, therefore being unphysiological for human ovarian tissue. If any cultured material is to be used for transplantation purposes, good manufacturing practice stipulates that all constituents of the culture system should not be of animal or plant origin.

Therefore, an alternative solution was offered by using collagen type I, which is a normal constituent of ovarian tissue. Collagen fibers are the most abundant protein constituents of the ECM, and various subtypes of collagens have been demonstrated in both the animal and human ovary [9, 53–55]. The ECM of the ovary is composed of a variety of molecules that are involved in a multitude of functional processes, including steroidogenesis and luteinization [51, 56, 57]. Whereas collagen type IV has been shown to be present in the basal membrane, separating the theca interna and the granulosa, and collagen type I and type III are present in the theca externa and type I between the individual granulosa cells [58]. Together with laminin, another important component of the basal membrane, collagen interacts with its neighbouring granulosa cells via integrins expressed on the membrane of GCs [48, 59].

It has now become possible to culture human GC over prolonged time periods in the presence of LIF, as set of experiments was designed to demonstrate that a significant subpopulation of human luteinizing GCs collected from mature ovarian follicles are able to maintain their functional characteristics over prolonged time period, when they are cultured in a 3D matrix made of collagen type I, and that they can become integrated into newly developing follicles after GC transplantation into the ovaries of immunoincompetent mice [60].

## 5. Granulosa Cells Phenotype in Accordance to Neofolliculogenesis

**Figure 1:**
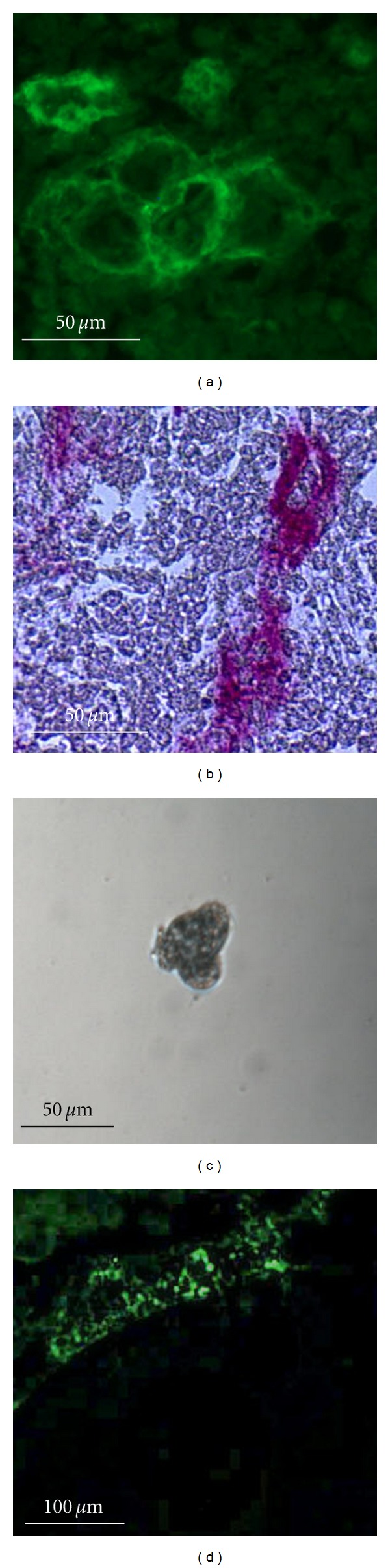
(a) Follicular cells cultured in 3D conditions together with type I collagen after 3 weeks (a) presented positive staining for FSHR (b) and positive staining for type IV collagen. (c) Clonogenic proliferation of follicular cells collected from mature ovarian follicles of infertile women treated with assisted reproduction and sorted with FACS based on the presence of the follicle-stimulating hormone receptor (FSHR). Clonogenic proliferation of a single follicular cell cultured for 12 days in a single well in the medium supplemented with leukemia-inhibiting factor (LIF). (d) Follicular cells cultured for 3 weeks in 3D conditions together with type I collagen were transplanted into the ovaries of immunodeficient mice. Immunostaining for HLA-ABC detected human cells after transplantation into immunodeficient mice observed in mice oviduct (data from previously published experiments in [39, 60]).

The 3D culture system of GCs coated with collagen type I not only extended cellular survival *in vitro* but also allowed GCs to maintain many of their morphological and functional characteristics such as FSHR, LHR, and P450 aromatase [60]. The demonstration of both FSHR and Coll IV in GCs cultured in 3D suggested that this culture system mimics physiological ovarian follicular development (Figure 1). Similar experiences were made earlier when whole ovarian follicles were cultured *in vitro* [9, 56, 61–64]. In a 3D culture system with intact murine follicles collagen type I promoted an increase in size of two-layered follicles but had no effect on multilayered follicles [9]. 

In the 3D model based on collagen type I in culture medium supplemented with LIF, Coll IV was found to surround patches of GCs containing the FSHR [60]. This is in accordance with the observation that in early antral follicles collagen type IV is localized specifically in the basal membrane [65], whereas in preovulatory follicles collagen type IV is also detected in more central layers of the granulosa [57]. The basal membrane influences GC proliferation and differentiation [62, 66–69], and above observation confirms previous results [55] by demonstrating that collagen type IV, a major component of the basal membrane and of the ECM, is produced by the GCs themselves.

Taking all these findings together, the GCs in the 3D culture system based on collagen type I and LIF display a development which is reminiscent to surrogate follicle-like structures. However, for ethical reasons the culture of human oocytes was not attempted systematically.

The proliferative potential of human GCs and their ability of long lasting when in culture with follicular fluid (FF) were recently confirmed [70]. There are many advantages of coculturing GCs with FF, which among other molecules contain LIF. Being the natural environment for GCs, FF retained their morphology and intercellular connections, improves their attachment in culture and proliferation of primary culture.

Additional proof of proliferation potential of human granulosa cells is the clonogenic growth of single GCs to 3D colonies, when they are cultured in the presence of LIF (Figure 1) [60].

## 6. Somatic Stem Cells in the Ovary

In contrast to the ongoing controversy with regard to the possibility of ongoing renewal of oogenesis in the ovary and the possible existence of oocyte-producing stem cells in the adult ovary, the existence of stem cells sustaining the other compartments of the ovary has long been neglected. 

Conventional thinking considers the ovarian follicle as an isolated structure, distinct both in space and time, either destined for early degeneration through atresia or for growth to a mature, Graafian follicle, ovulation, and formation of the corpus luteum. The cyclicity of follicular development, ovulation, and luteal function is seen as discrete phenomena, separated both in time and space. In recent years, however, experimental evidence has shed some doubt on this conventional thinking. Most notably, it has been demonstrated that mature and fully grown mouse oocytes are able to influence the development of preantral follicles in mouse ovaries [71], indicating the interdependency of the cyclic events occurring during subsequent menstrual cycles. In addition, other investigators were able to demonstrate that upon ovulation, the epithelioid granulosa cells redifferentiate into the mesenchymal cells of the corpus luteum [2].

GCs cultured *in vitro* invariably cease to proliferate already after two to three passages. Similar results have been obtained when culturing thecal cells. Recently, it has been shown that the four growth factors, bFGF, EGF, LIF, and IGF1, exhibited significant enhancing effects on colony growth of thecal cells, leading to the detection of thecal stem cells in the ovary [71, 72].

A subpopulation of mural granulosa cells has now been demonstrated to contain cells with multipotent stem cells potential, as they express the stem cell marker Oct-4 [39]. POU5F1 (Oct-4) is known to be expressed in human epithelia [73]. As GCs represent one of the most dynamic epithelium in the body, this could be as well true for GCs and explain its stable expression of Oct-4. Functional properties of stem cells in general include pluripotency, mitosis without differentiation, when confined to their niche, and by their ability to proliferate when isolated out off their niche [74]. The ECM is crucial for maintaining the pluripotency of stem cells through contact inhibition. Lack of contact inhibition occurs *in vivo* during growth of a follicle [75] particularly during the preantral stage of follicular development, when granulosa cells are in close physical contact to the ECM [3]. 

The hypothesis for the presence of stem and progenitor cells in the granulosa was first postulated, when it was demonstrated that the granulosa possesses some marked similarities to other epithelia in the body [2, 67]. The granulosa of ovarian follicles resides on a basal membrane, and the morphology of the cells highly differs in various regions of the granulosa. These authors concluded that there must be populations among the granulosa cells containing less differentiated cells and, at a distance from these, populations with highly differentiated cells.

**Figure 2:**
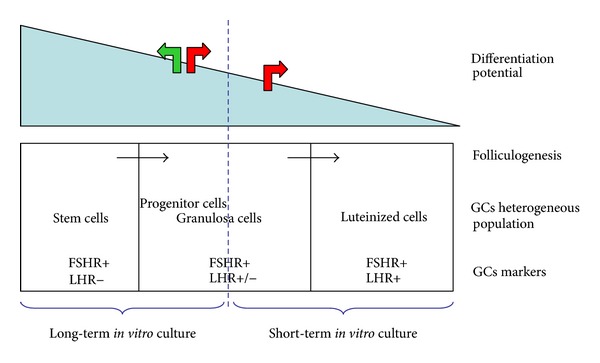
Hypothesis presenting several subpopulations of GCs within preovulatory follicle. GCs stem cells: positive only for FSHR. Progenitor GCs: positive for both FSHR and moderately for LHR markers. Luteinized GCs: positive for FSHR and LHR markers.

Most adult tissues contain a heterogeneous population of cells with a hierarchy of multipotent stem cells, progenitor cells, and terminally differentiated cells [76]. We hypothesized that within preovulatory follicle there are several subpopulations of GCs with distinct characteristics. GCs expressing both FSHR and LHR, denominated luteinized GCs, will enter apoptosis during prolonged culture *in vitro*. GCs, positive for FSHR but not for LHR, may either become dedifferentiated to the progenitor GCs or differentiate to luteinized GCs (Figure 2).

It was suggested earlier that the cohort of granulosa cells in a human preovulatory follicle is derived from a clonal expansion of a small number (3 cells) of ovarian stem cells [77]. The presence of a subpopulation of GCs with multipotent stem cell characteristics explains why GCs, taken from preovulatory follicles and cultured under the appropriate conditions, can survive over prolonged time periods and can be differentiated into other tissue types, otherwise not present in the ovary. In addition, when cultured in 3D in an extracellular matrix similar to the ovary, the GCs seem to retain most of their characteristics including the FSHR and steroidogenesis [60].

In addition to the granulosa cells FSHR/LHR subgroups the substantial subpopulations of long cultured granulosa cells were attained by expression of mesenchymal lineage marker CD117 (c-kit). The results differed within patients (Kossowska-Tomaszczuk and coworkers; data unpublished).

GCs stained for both FSHR and CD117 (c-kit) established 3 populations: cells positive only for FSHR, cells positive for both markers, and cells negative for those markers. Our observation showed that women with good quality of oocytes and the one who got pregnant after the IVF therapy had higher amounts (25%–40% of total GCs) of cultured GC positive for both CD117 and FSHR markers than a population only positive for FSHR or with none. However, correlation between the number of those cells among fertile and subfertile women failed to indicate any role with respect to the likelihood of successful pregnancy.

c-Kit is the stem cell factor receptor and is expressed in human ovarian follicular development, and their interaction is required for the survival of follicles in long-term culture [78]. c-Kit was confined to the oocyte and granulosa cells in primary and secondary follicles and preovulatory granulosa cells [28, 79]. 50–70% of freshly isolated granulosa cells from patients undergoing assisted reproduction contained cells which stained positively for c-Kit [80]. c-Kit signalling is likely to control the survival of human ovarian follicles during early follicular development. Blocking the c-Kit receptor induces follicular atresia.

## 7. Conclusion: The Potential Significance of Granulosa Stem and Progenitor Cells 

Together with the stem cells located in the theca [72, 81], the multipotent stem cells of the progenitor type in the granulosa [39] may provide the niche, in which oocyte-producing stem cells may thrive [82]. The finding of granulosa cells displaying multipotency and having a prolonged lifespan, extracted from mature ovarian follicles, is likely to have a significant impact on evolving theories in ovarian physiology and reproductive biology, particularly with reference to folliculogenesis and the pathogenesis of ovarian endometriosis and ovarian cancer. Multipotent stem cells in ovarian follicles may be involved in the early origin of some forms of ovarian tumors, in particular granulosa cell tumors, as well as to the origin of ovarian endometriosis, which is considered to arise from undifferentiated, hitherto labeled as metaplastic cells in the ovary (Figure 1). 

In female mammals, the normal and physiological production of good quality gametes relies upon the highly controlled growth and differentiation of the surrounding ovarian follicle. GC proliferation is maintained throughout folliculogenesis, providing not only a specialized microenvironment but also nutrients for oocytes growth. GC multipotency and the use of granulosa stem and progenitor cells in the newly developed 3D *in vitro* culture system may provide a promising technical tool for *in vitro* maturation of both human and animal ovarian follicles. Such culture systems can be used in reproductive toxicology, in drug targeting, and in assisted reproduction, respectively, breeding.
